# Multidisciplinary Approach Applied to the Diagnosis of the Facade of the Arciprestal Church of Santa María de Morella (Castellón, Spain)

**DOI:** 10.1155/2019/2852804

**Published:** 2019-05-07

**Authors:** Livio Ferrazza, María T. Pastor Valls, Gemma M. Contreras Zamorano, David Juanes Barber, Roxana Radvan, Alexandru Chelmus, Lucian Ratoiu, Luminita Ghervase, Ioana Maria Cortea, Pilar Ortiz

**Affiliations:** ^1^Instituto Valenciano de Conservación, Restauración e Investigación (IVCR+I), Valencia C/Genaro Lahuerta 45-3°, 46010, Spain; ^2^INOE 2000, Str. Atomistilor Nr., 409 Magurele, Ilfov, Romania; ^3^Departamento Sistemas Físicos, Químico y Naturales, Universidad Pablo de Olavide, Sevilla, Ctra. Utrera Km1, 41013, Spain

## Abstract

This paper deals with the development of a multidisciplinary study on the current state of conservation of the facade of the Arciprestal Church of Santa María de Morella (Castellón, Spain), a work of the Gothic period of great historical and artistic value. The aim of this diagnosis was to undertake the preventive conservation actions required and increase the knowledge about the conservation of paintings on stones. During the diagnosis scanning, electron microscopy was demonstrated to be a valuable analytical method for wall paintings on stone. The facade, which since its construction has not undergone major architectural changes, has reached our days as it was configured in its creation, adding the traces of the passage of time and interventions that have suffered polychromies. Because of the conservation situation, it was decided to have an interdisciplinary project for the structural study of the work, an exhaustive study of the materials and their state of conservation. The study of the materials includes the identification of stone supports, mortars, the pictorial technique of the original and added polychromies, and the superficial patinas. On-site studies were carried out by ground penetration radar (GPR) and X-ray fluorescence (XRF). Among the techniques used in laboratory were optical polarized light microscopy (MO-LP), X-ray diffraction (XRD), scanning electron microscopy with microanalysis (SEM-EDX) and Fourier-transform infrared spectroscopy (FTIR). The study allowed to determine the different pathologies of alteration and degradation of stone substrate and polychromies, chromatic alterations, biological patinas, etc. During this study, it was demonstrated that the diagnosis of wall paintings is a complex issue that needs to be addressed in a multidisciplinary approach, where scanning electron microscopy with microanalysis is the key methodology to get a deeper understanding of subsurface characterization of wall paintings and highlight the weathering processes. In a second phase of previous studies, this technique (SEM) has been used in assessing the viability of consolidation systems and cleaning both the stone and the polychrome.

## 1. Introduction

SEM-EDX is widely employed for wall painting and polychromies in cultural heritage, though is commonly combined with other techniques [1–5]; in most cases, SEM-EDX is the key technique that allowed to take decisions on the restoration of wall paintings, polychromies, and stone surfaces. Moreover, SEM-EDX allowed to validate the results of other techniques employed for paintings [6, 7]. In this paper, a multidisciplinary approach applied to the restoration of the facade of the Arciprestal Church of Santa María de Morella (Castellón, Spain) is shown to evaluate the relevance of SEM-EDX in the decision process of restoration of wall paintings.

The Archpriest Church of Santa Maria was built in Gothic style, during the thirteenth and fourteenth centuries. The facade has two main doors of special beauty: most known as the Apostles (XIV century) and the second call of the Virgin (XV century). It highlights the fact that both doors are on the same side and one is beside the other.

**Figure 1:**
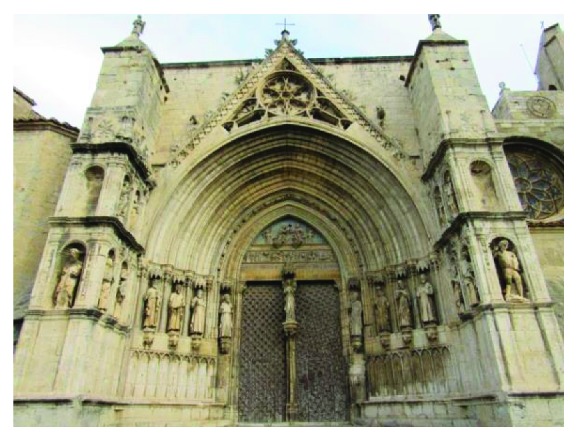
Door of the Apostles, Archpriest Church of Santa Maria of Morella.

The Door of the Apostles (dels Apòstols) is the cover object of the interdisciplinary study (Figure 1). The portal is named by the apostolate that is housed in the bottom of the blind arches and jambs. The eardrum has a frieze run where scenes from the life of Mary and childhood of Jesus are represented. We also found a Madonna and Child under a Gothic canopy in the mullion of the door. In the tympanum, there is a representation of the Coronation of the Virgin. Besides this door, it has a Gothic window and a rose window that illuminates the interior of the Church [8].

The Archpriest Church of Morella was declared of Cultural Interest in 1931: Erected Minor Basilica by Pope Pius XII (between 1939 and 1958). In 1402, Pope Benedict XIII granted the privilege of asylum. Built in Gothic style between 1265 and 1343, it was consecrated in 1318 in the presence of the King of Aragon Jaime II the Just (1291-1327). Its main facade has two doors of special beauty: most known as the Apostles and the second call of the Virgin. It highlights the fact that both doors are on the same side and one next to the other, thus constructed by the need to adapt to the ground and avoid the embankment of the castle.

## 2. State of Conservation

**Figure 2:**
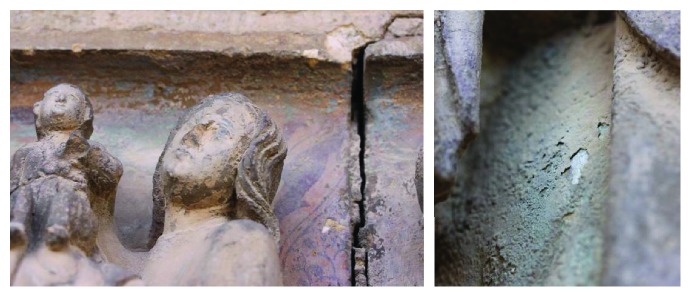
Door of the Apostles, Archpriest Church of Santa Maria of Morella. Details of the state of conservation of the polychromy on stone.

With regard to the conservation status of the door, it was observed during the diagnosis that the pathological processes, more or less important depending on the area studied, are related to the degradation of stone material and polychromies. The images in Figure 2 are details of the frieze on the door, where you can see the important alteration of the stone and the polychromies, with disintegration mechanisms, surveys, losses, and superficial deposits.

**Figure 3:**
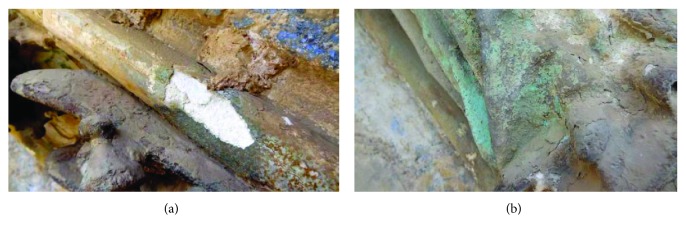
Details of the Door of the Apostles, Archpriest Church of Santa Maria of Morella. (a) Deposits of dirt, formation of crusts, and decohesion stone support. (b) Details of dirt, crusts, and areas with lifts and loss of cohesion of layers.

The alterations that the door presents are varied and have introduced changes in the stone at a superficial level and in the polychromies. In the latter case, the color layers have obviously suffered mechanisms of alteration due to dustiness due to loss of binder, desquamation, formation of surface crusts, manifesting in differential chromatic tones, changes in surface texture, and variation in chemical composition and physical properties (Figures 3(a) and 3(b), details of the sculptural elements on the door). The chromatic alterations are easily observable and are mainly due to deposits of dirt and possible chromatic alterations of the pigments.

Therefore, considering the situation that the elements make up the cover, it was decided to have an interdisciplinary study project necessary to the future restoration that would involve an exhaustive study of the stone support and mortars, the characterization of the original pictorial materials and additions, and the identification of the surface patinas.

## 3. Materials and Methods

### 3.1. On-Site Studies

Using ART4ART [9], INOE's mobile laboratory, a program of smart investigations was conducted on the façade of the Arciprestal Church of Santa María de Morella; its purpose was not only to obtain the maximum possible amount of information but also to lead a quantitative evaluation of potential degradations.

The infrastructure used covers a wide range of equipment that can be divided into elementary physicochemical analysis and ground penetration radar (GPR). Elementary physicochemical analysis was used for material identification while ground penetration radar (GPR) is a noninvasive geophysical method used for in depth investigations by emitting and studying the propagation of electromagnetic pulses, to highlight the heterogeneity or discontinuities of the electrical properties of the propagation medium. This technique is mostly used on the soil surface but can also be used on the vertical acquiring way of information about the internal structure of the walls or their state of preservation by noncontact, noninvasive, nondestructive means.

Elementary composition was studied on-site by XRF equipment TRACER III-SD with Rh tube, standard (Bayes) methods (40 kV, 10.60 *μ*A), and an acquisition time of 10 s/spectrum. XRF analyses have been performed on 14 areas: (a) nine points on the 13th century area (scarlet, green, blue, and substrate), (b) one point on the 14th century area (scarlet, substrate), and (c) four points on the 15th century area (ochre, scarlet, and blue).

For the vertical radar investigations conducted on the portal, a GPR antenna having an 800 MHz central frequency with the electromagnetic pulse travel times set at 40 and 20 ns was used. For the data interpretation the relative dielectric permittivity of 9 was considered, resulting this way an approximatively 2-meter penetration depth for the 40 ns travel time and a 1-meter penetration depth for the 20 ns. The purpose of this investigation was to observe how deep are the three visible cracks present on the east side of the portal, above the access door, and also to identify other nonvisible cracks of the wall, for conservation status evaluation. All the records were taken from west to east to obtain comparative results. For data interpretation, the west part will be used as a reference, due to nonvisible cracks.

### 3.2. Laboratory Studies

The successive phase of the study focused on analyzing samples corresponding to fragments of the stone support, mortars, the materials present in the polychrome layers, and the alteration products. The samples were analyzed by the following techniques:

- The identification of the organic compounds and the different crystalline phases was carried out by IR analysis with infrared spectroscopy with Fourier transform infrared spectroscopy (FTIR) of Bruker Corporation Tensor II, with a range of 4000-400 cm^−1^ with a resolution of 4 cm^−1^
- Polarized light optical microscopy (PL-OM) was performed using the ECLIPSE 80i microscope from Nikon Corporation that has a DS-Fi1 camera attached. The samples prepared in cross section and in thin sheets with a thickness of 20 *μ*m were observed in visible and ultraviolet light reflected and transmitted
- Scanning electron microscopy was performed using the variable pressure scanning electron microscope model S-3400N from Hitachi Ltd. (VP-SEM), equipped with an energy-dispersive X-ray spectrometer (EDX) from Bruker Corporation XFlash®. The working conditions were as follows: acceleration voltage of 20 kV, measurement time between 30 and 100 s, and working distance of 10 mm

## 4. Results and Discussion

### 4.1. On-Site Analysis

Due to the distance between the GPR antenna and the stone when the records were conducted, from 0 to 5 ns, we have a homogeneous response which represents the air that is why in all the radargrams, the first reflection was obtained at approximately 5 ns, which represents the surface of the stone. Other reflections that can also be seen in all the radargrams are the one at a 45° that are present both in the left and in the right sides of the records which were produced due to the sculpture presence on the edges of the investigated area; this reflection is highlighted with red at different travel times (Table 1).

In the radargrams, the horizontal axis represents the length of the record, and the vertical axis represents the travel time of the electromagnetic wave through the propagation medium (in our case stone), measured in ns. Both in the 20 ns and in the 10 ns radargrams conducted on the East side of the portal (581, 586), hyperbolic reflections can be observed which represent the crack; due to the strong reflection, it can be estimated that the crack goes in depth. In the rest of the radargrams acquired on the East part of the portal, the same results could be observed in the crack areas. On the stone block surface or in depth, any other cracks cannot be observed; then, the three ones were visible on the surface, representing the main structural problem on the stone facade.

XRF studies show the lines of Au on the 13th and 15th century areas on the scarlet areas. As and Co lines have been evidenced on blue areas; meanwhile, Cu lines were seen on blue, green, and scarlet. Fe is found at higher intensities on scarlet, ochre, and green. Hg lines are evidenced on scarlet, blue-scarlet, blue, and green zones. K lines are present on blue, scarlet, green, and ochre and on the substrate. Mn is present on ochre, blue, scarlet, and green. Sporadic content of barium and trace of Cl were detected in different points.

Those results and the state of conservation on painting layers highlighted the necessity of a SEM-EDX study in order to understand the degradation process on the painting of stones.

### 4.2. Laboratory Study

#### 4.2.1. Stone Support: SEM-EDX Study

**Figure 4:**
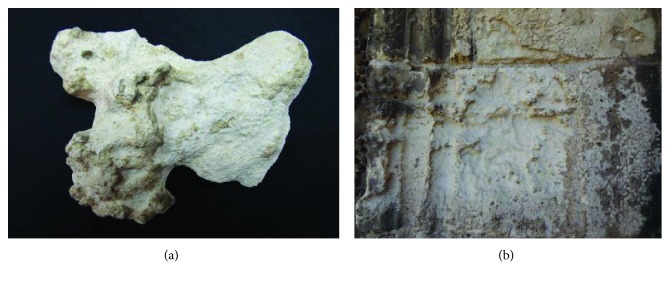
Detail of an ashlar stone of the Door of the Apostles, Archpriest Church of Santa Maria of Morella. (a) Conservation status of the stone support and (b) extracted fragment for a laboratory study with SEM-EDX.

Different fragments of stone support were extracted from the door, in areas significantly affected by alteration mechanisms with disintegration and pulverization phenomena (see Figures 4(a) and 4(b)). The main instrumental technique used to assess the state of conservation of the stone has been the SEM-EDX [1, 10]. Through this technique, it has been possible to observe and analyze different surface areas of the stone, without previous preparation and in cross section.

**Figure 5:**
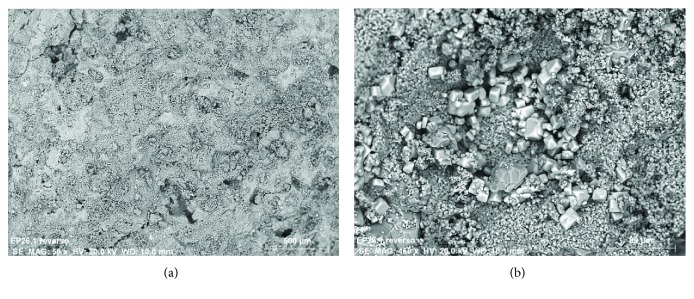
SEM micrographs in backscattered electron mode. (a) General morphological characteristics of the surface of the stone and (b) crystallizations of salts.

**Figure 6:**
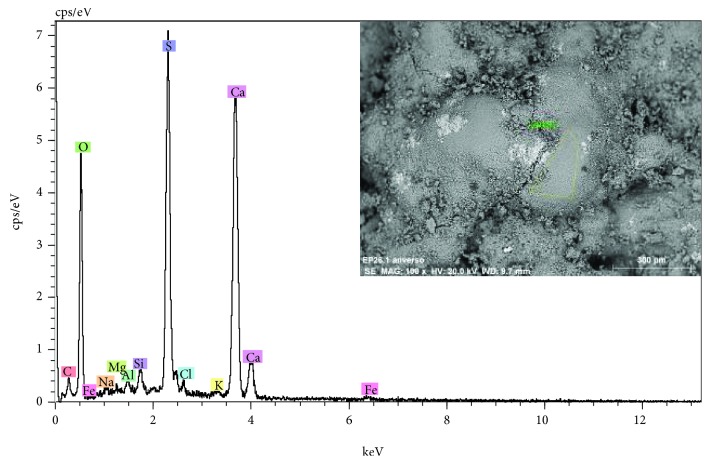
EDX microanalysis and SEM image (inset) of an area of the surface of the stone. The elements of calcium and sulphur corresponding to the presence of calcium sulphate.

**Figure 7:**
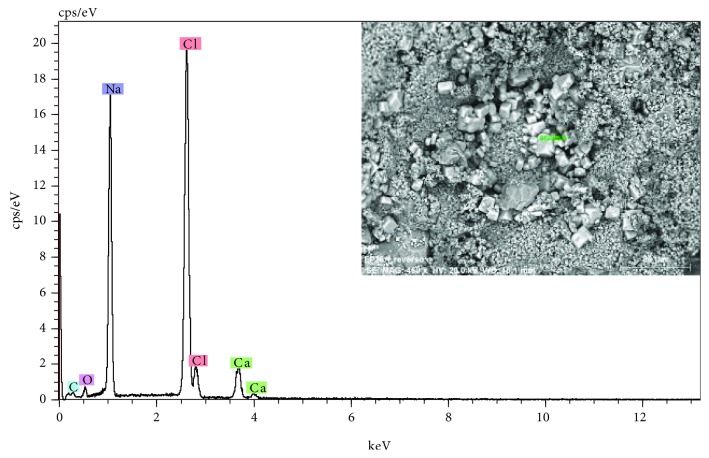
EDX microanalysis and SEM image (inset) of salt crystallizations. The elements of chlorine and sodium corresponding to sodium chloride.

The observations to the stone surface by SEM show a rather eroded structure, with a generalized presence of disintegration, fracturing, increased porosity, and crystallizations of products of different nature (see the SEM micrograph of Figure 5(a)). The microanalyses detect high concentrations of the chemical elements of calcium, with lower proportions of silicon and aluminum. The chemical elements can be associated with the calcium carbonate CaCO_3_, the main constituent of the stone support and mineralogical compounds based on aluminosilicates. Generally, on the surface of the stone, sulphur related to calcium sulphate CaSO_4_ is detected as a product of chemical alteration of calcium carbonate or as crystallizations due to the presence of saline aqueous solutions (see spectrum of the EDX microanalysis of Figure 6). As shown in Figure 5(b), the surface presents characteristic crystallizations of cubic morphology, which based on the EDX microanalysis are constituted by chlorine and sodium, indicating the presence of salts based on sodium chloride (see Figure 7). The crystallizations of the different salts, in the interior and in the surface of the stony substrate, would cause part of the mechanisms of disintegration and pulverization of the microcrystalline structure of the rock [11].

**Figure 8:**
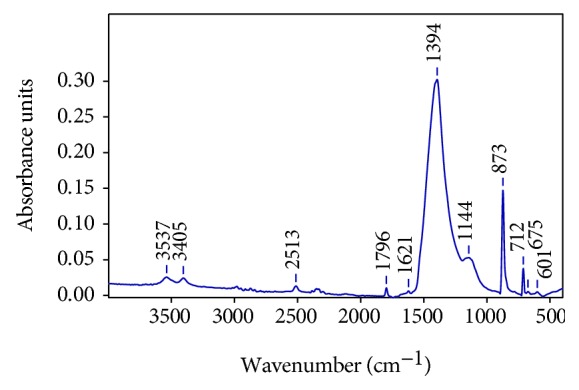
FTIR spectrum of the sample surface of the stone support.

The identification of the mineralogical components has been carried out using FTIR. The analyses have confirmed the presence of calcium sulphate in its dehydrated phase CaSO_4_·2H_2_O (gypsum) and calcium carbonate (CaCO_3_, calcite) as a constituent of the stone. In the spectrum of Figure 8, the characteristic absorption bands of calcium carbonate at 1420 cm^−1^, 875 cm^−1^, and 712 cm^−1^ and calcium sulphate dihydrate at 1144 cm^−1^, 675 cm^−1^, and 601 cm^−1^ are observed. The displacement of the sulphate SO_4_^2-^ band at 1394 cm^−1^ is possibly due to sodium chloride.

**Figure 9:**
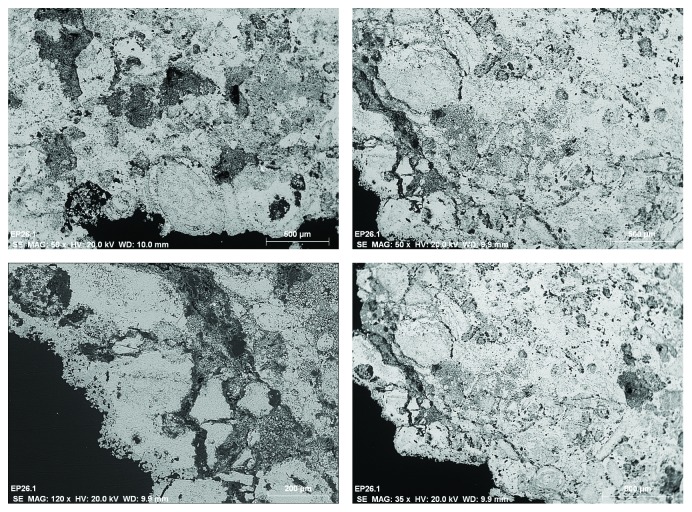
SEM micrographs in the backscattered electron mode of different areas of the cross section of the stone support.

**Figure 10:**
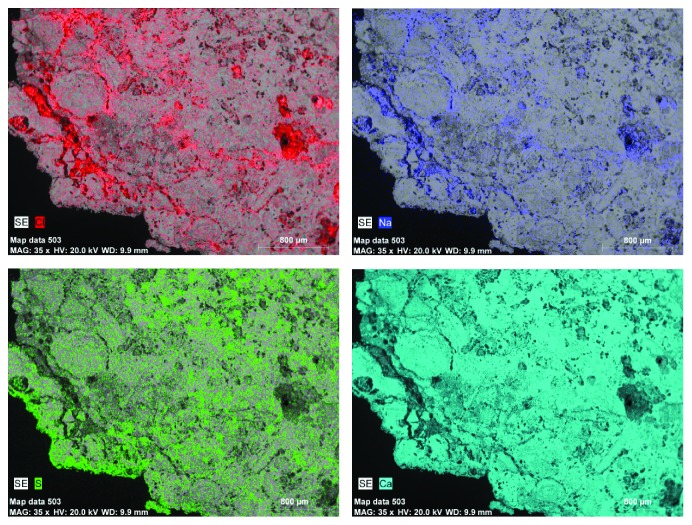
Distribution map of the elements of chlorine, sodium, sulfur, and calcium in the cross section of the stone support.

The stone support has been successively observed and analyzed in cross section [12]. The stone is characterized by high porosity and microfractures due mainly to the internal and superficial crystallization of salts as can be seen in the backscattered electron mode of SEM micrographs (Figure 9). In the X-ray map of Figure 10, the chemical elements of chlorine, sodium, and sulphur, related to the presence of sodium chloride and calcium sulphate in the structure of the stone, are identified and located. Calcium is related to both calcium sulphate and calcium carbonate, constituents of the stone support. The maps evidence that halite and gypsum are weathering forms and halite occupied mainly the pores and factures under the surface while gypsum is located on surface and inner pores. The distribution of both alteration products occupied different positions on the structure of the stone, and their combined action is the cause of the weathering forms observed due to the formation of surface crusts and desquamation.

Generally, high concentrations of sodium chloride are detected in the lower areas of the cover, possibly due to the treatment of the soil with salt in the presence of snow. Differently, the presence of calcium sulphate is quite generalized; this last compound can be related both to the mechanisms of chemical alteration of the stone support and to superficial deposits.

The results provided by the SEM-EDX, through observations and microanalysis of stone samples, will allow establishing the most appropriate intervention methodologies for the work, such as the elimination of soluble salts or consolidation treatment support in the areas of greatest disaggregation.

#### 4.2.2. Polychromy Study

**Figure 11:**
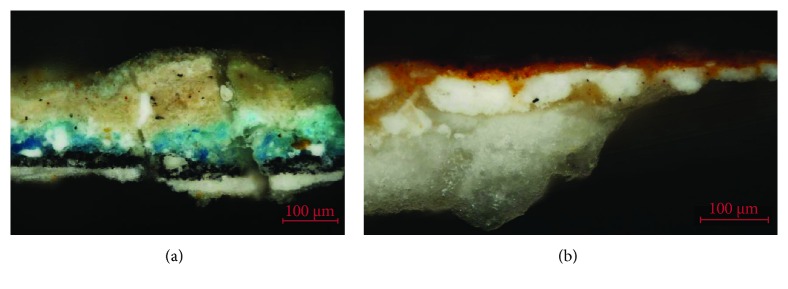
Optical microscopy images. (a) Stratigraphic section of a blue polychrome sample. (b) Stratigraphic section of a sample of ochre polychrome.

The polychromatic samples have been prepared in a stratigraphic section for their study with OM with visible and ultraviolet light and SEM-EDX [13, 14]. The techniques have identified in the primitive intervention of the fourteenth century, where there is existence of a white preparation stratum that sits directly on the stone support, followed by the different layers of color. In general, a polychromy is observed with a careful and rigorous traditional technique where there is no complex superposition of layers, in which the general thickness of the color layer ranges between 15 and 30 *μ*m [15]. In the hair, garments, or decorative elements of the different sculptural elements, the gold leaf has been found on a reddish preparation based on earth, demonstrating the existence of a wide decoration of the cover with gold elements. The images in Figure 11 are examples of the stratigraphic study with optical microscopy of samples corresponding to the oldest polychrome intervention on the door.

**Figure 12:**
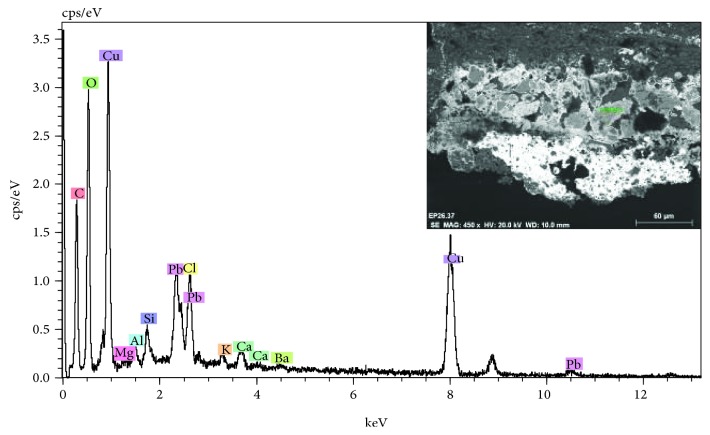
EDX microanalysis and SEM image (inset) of the blue pigment present in the stratigraphic section. The copper characteristic of azurite is detected.

The pigments have been characterized with SEM-EDX by identifying the characteristic chemical elements. These have been related to the pictorial materials used, which turn out to be typical of the period such as lead white, yellow ocher, red ocher, earth, azurite, copper green, and carbon black.  Figure 12 shows the microanalysis performed on a blue particle present in the polychrome layer, in which copper is detected, a chemical element that is related to the azurite pigment (Cu_3_(CO_3_)_2_(OH)_2_). In the same microanalysis, the high chlorine content can be seen that corresponds to the presence of salts or a possible chemical alteration of the pigment. In general, the pigments are finely ground, with the exception of the azurite pigment, which is observed with a coarser granulometry, since it is a less opaque pigment. In the latter case, the blue layer of azurite sits on a layer of carbon black preparation (see Figure 11(a)). These results confirm that in the old polychrome of the door of *Los Apóstoles*, the use of a technique and pictorial materials that are common in the XIV-XVI centuries is reflected [16].

**Figure 13:**
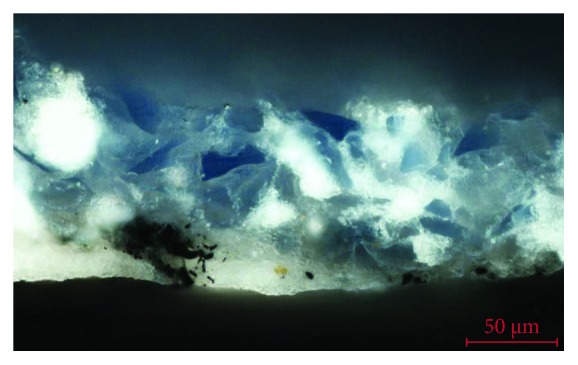
Optical microscopy image of the stratigraphic section of a blue polychrome sample corresponding to an intervention.

**Figure 14:**
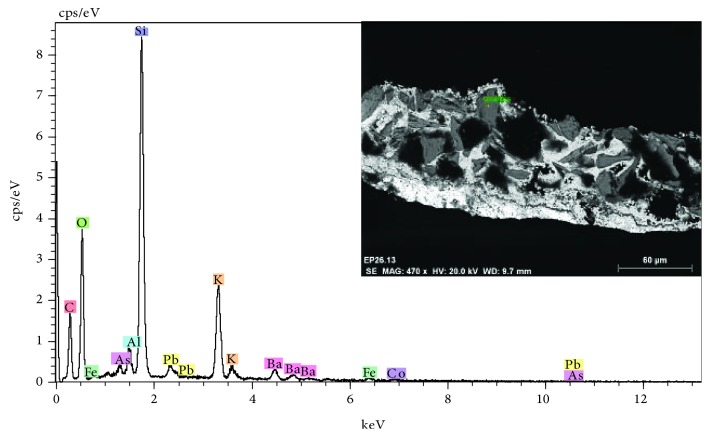
EDX microanalysis and SEM image (inset) of the blue pigment present in the stratigraphic section. The elements of silicon and cobalt characteristic of blue enamel are detected.

The study has also made it possible to identify the subsequent polychromatic interventions carried out on the cover. In this case, specific interventions have been observed in the sculptural elements, where the most characteristic is the use of the blue enamel pigment (cobalt silicate), according to XRF data. A pigment of artificial origin is known since the middle ages in the processing of glass. In painting, it was used during the XVI and XVII centuries [17] (see Figure 13 and the microanalysis spectrum of Figure 14).

**Figure 15:**
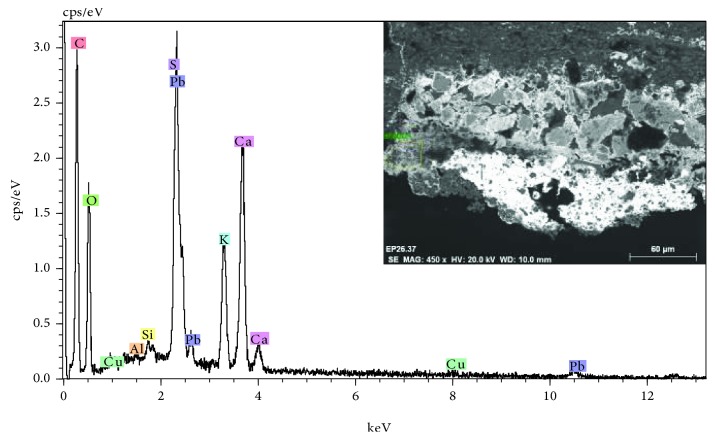
EDX microanalysis and SEM image (inset) of an area corresponding to the alteration of the white lead pigment. A high concentration of potassium is detected.

**Figure 16:**
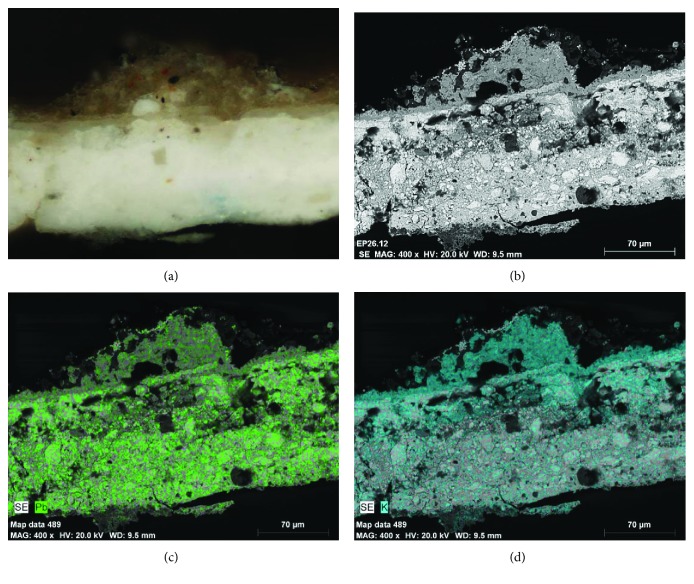
(a) Stratigraphic section of the polychrome sample observed with optical microscopy and (b) respective SEM image in the backscattered electron mode. (c, d) Distribution map of the elements of lead and potassium.

The stratigraphic study with SEM-EDX has been decisive in evaluating the conservation status of the color layers and their constituents. As shown in the EDX microanalysis of Figure 15, lead white is usually found associated with high concentrations of potassium and sulfur. This result is possibly due to the chemical alteration of the pigment where among the possible causes, an intervention of cleaning the surface with potassium carbonate can be considered. The treatises of the 20th century advised this basic substance in solution with water to clean the dust deposits of the polychrome surfaces [18]. It is observed that, generally in an extensive way, this chemical alteration of the pigment is based on lead, with its possible solubilization, diffusion, and precipitation both in internal and superficial areas. The alteration of the lead white by the presence of potassium is evidenced in the optical microscopy image and distribution map of elements of Figure 16. Figure 16(a) is relative to the stratigraphic section of a white polychrome sample observed with optical microscopy. A first stratum based on lead white is observed, on which a layer of ocher shade is spread, which based on the EDX microanalysis is made up of elements of dirt and alteration products enriched in potassium.

It is quite frequent to find in stone polychromes the alteration of mineral pigments based on lead or copper, due to environmental factors such as humidity or the presence of salts, as well as cleaning interventions with chemical systems or biocide treatments. Recent studies have shown how the polychrome treatments with chlorinated substances can produce chemical and physical alterations to the white lead pigment with the formation of compounds based on lead hydroxychloride [19].

**Figure 17:**
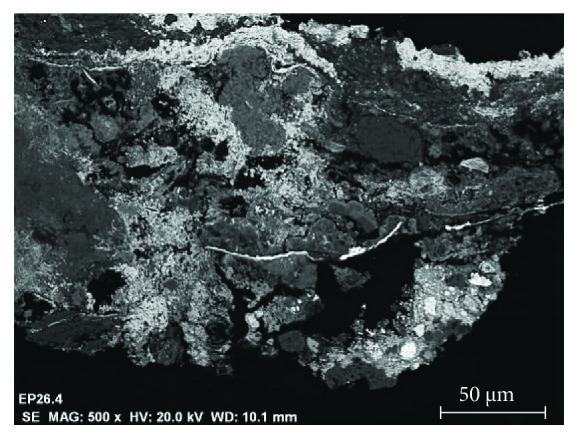
Stratigraphic section of a polychromatic sample observed at the SEM in the backscattered electron mode.

The majority of the samples present a polychrome with a disintegrated and pulverulent appearance. This alteration is due to the partial loss of the binder, as well as the crystallizations of sodium chloride and calcium sulphate that produce a series of longitudinal and/or transversal ruptures that cause both a lack of adhesion and cohesion of the strata, resulting in detached layers (see the SEM micrograph of Figure 17).

**Figure 18:**
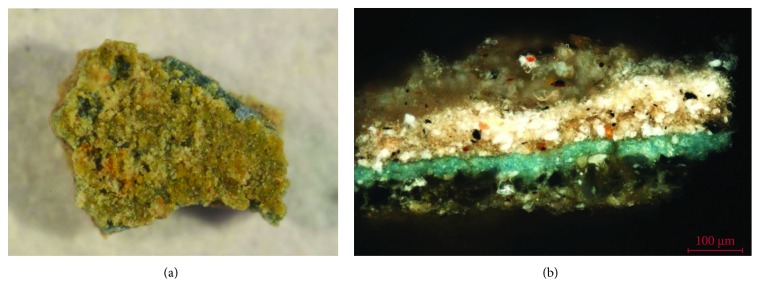
(a) Blue polychrome sample with an ocher-colored superficial crust. (b) Stratigraphic section of the polychrome sample observed with optical microscopy.

**Figure 19:**
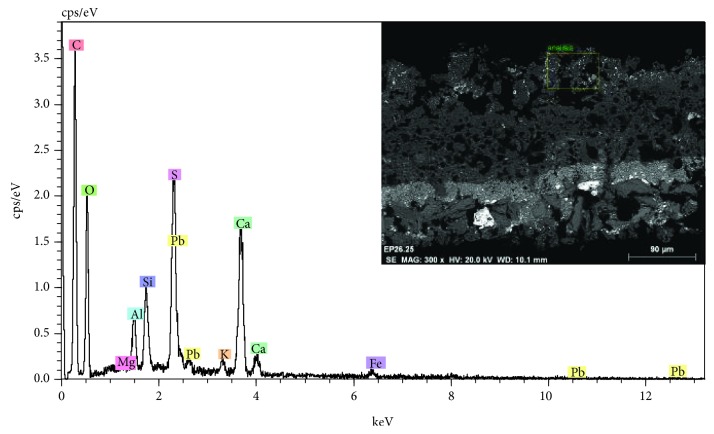
EDX microanalysis and SEM image (inset) of the stratigraphic section of a sample with a surface crust. The calcium, sulfur, silicon, and aluminum elements that are associated with the presence of calcium carbonate, calcium sulfate, and aluminosilicates are detected.

Another significant aspect is the presence of a crust of ochre huge on the surface of the polychrome. In the stereoscopic microscopy image of Figure 18(a), relating to a sample of polychrome extracted from the door, the presence of a surface layer of ochre tonality that extends over the blue polychrome is observed. As shown in the images of the stratigraphic section observed with optical microscopy (Figure 18(b)) and electron microscopy (Figure 19 inset), this stratum has a considerable thickness and is basically composed of calcium and sulphur, with varying concentrations of silicon and aluminum, as reflected in the microanalysis spectrum of Figure 19. The characterization of the mineralogical constituents carried out with FTIR has identified the presence of calcium sulfate, calcium carbonate, silicates, and calcium oxalate monohydrate (Whewellite).

All the damages detected are due to physical-chemical processes, including those of a biological type, as well as an anthropogenic type [20, 21] that have been clearly unveiled by SEM-EDX. Of course, all these processes may act synergistically and depend on climatic/environmental conditions and atmospheric pollutants and certain human activities. Among the processes that are damaging the door are the processes of alteration by thermal shock, condensation, frost, repeated cycles of crystallization, and recrystallization of salts and biological attack; these last four are directly related to the presence of water (and its pollutants) detected by SEM-EDX in its different variables and penetration via the affected surfaces [22–25].

The processes detected, added to the architectonic design with numerous exempt and cantilevered elements, made a quick intervention necessary in order to avoid the progress of the existing alterations that would lead to the state of ruin, already initiated in a large part of the elements, and it is evidenced in the periodic detachment of carved stones.

SEM-EDX mappings allow to get the overall objective of this work that was to document the conditions of conservation and the causes of deterioration of the materials, determining the different pathologies of chemical, physical, and biological alteration [26, 27]. Though a multidisciplinary approach is always advisable in the diagnosis of cultural heritage, SEM-EDX has been demonstrated in this work to be the key solution for the study of painting layers as it has allowed to identify sodium chloride, calcium sulphate, and an alteration of the white lead pigment associated to potassium as the main agents. The results allowed to define the most suitable methodologies to carry out direct and indirect interventions on the door [28]. This phase is necessary to eliminate or limit the influence of environmental factors on degradation processes, including conservation interventions and protection of architectural structures and decorations.

#### 4.2.3. The Application of SEM-EDX in the Evaluation of Conservation Treatments

**Figure 20:**
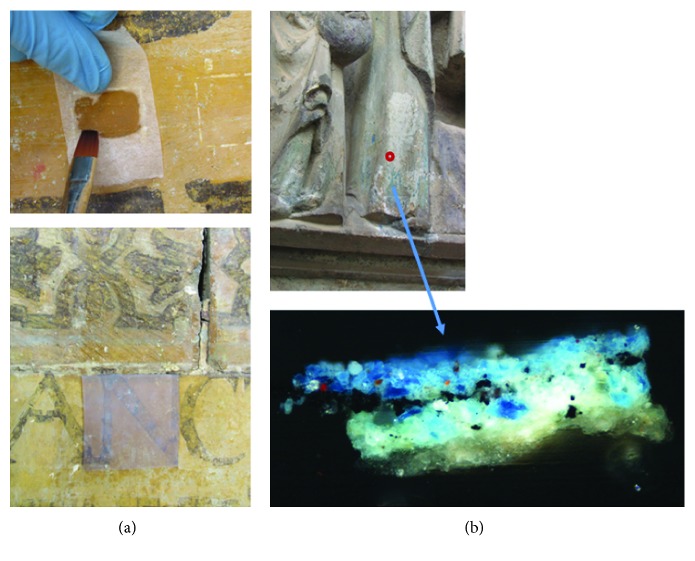
(a) Application of consolidants and agar agar gels for cleaning surface dirt. (b) Application of the laser as a cleaning and evaluation system using optical microscopy and SEM-EDX.

Although this article focuses on the application of the SEM-EDX technique in the diagnosis of the state of conservation and characterization of the materials used in the door, in a second phase of previous studies, this was applied in the evaluation of the viability of the cleaning and consolidation treatments of stone supports and polychrome. This microanalysis technique was very useful when establishing the most appropriate intervention methodologies, such as the effectiveness of the elimination of soluble salts through the use of packings, the penetration of the consolidants applied in the disaggregated zones, or the correct disposition of the applied polymers with adhesive function between adhesive layers. At the same time, its application in the monitoring of the various cleaning systems tested (agar agar gels, packaging, mechanical cleaning, or laser) stands out (Figure 20). Regarding the latter, the possible risks could be determined in terms of physical-chemical modification of the materials constituting the cover.

In this case, the evaluation of the treatments was carried out by means of sample extraction and its study with optical microscopy and SEM-EDX.  Figure 20(a) shows the stratigraphic section with optical microscopy of a blue polychrome sample after the laser cleaning treatment. The treatment allows the removal of surface dirt without affecting the integrity of the azurite polychromy.

## 5. Conclusions

This multidisciplinary approach highlights the necessity of SEM-EDX on pigments over stones. The analysis of the samples from the door of *Los Apóstoles* of the Basilica Arciprestal de Morella with scanning electron microscopy (SEM), complementing the studies with EDX microanalysis and other combined techniques, has revealed the conservation degree of the stone and pigment materials.

In detail, with the possibilities offered by the SEM-EDX, the mechanisms of alteration of chemical and physical type that significantly affect the future conservation of the stone support and the components of the polychrome layers such as the pigments could be studied. The observations and the microanalyses allow identifying the chemical alterations of the pigments, dissolution processes, migration and precipitation of soluble salts, and mechanisms of structural alteration of stone materials.

Studies with portable analysis techniques, without sample extraction, have been equally necessary to determine on a large scale the state of conservation of the materials (stone and pigments) and the structural damage that occurs in the different architectural elements of the door.

## Figures and Tables

**Table 1:** Radargrams of the façade. Measurements labelled as 569 and 575 at West zone and Measurements labelled as 581 and 586 at East Zone. Sculpture presence is highlighted with red at different travel times.

West (reference)		East	
569	575	581	586
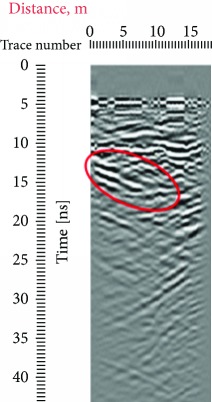	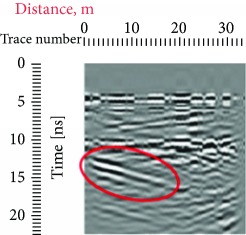	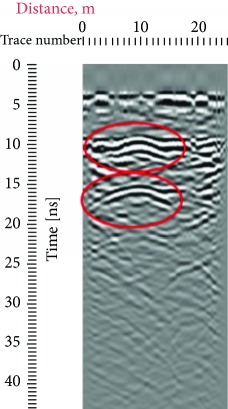	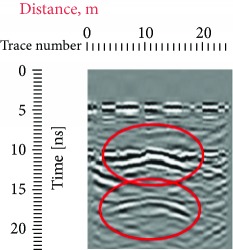

## Data Availability

No data were used to support this study.
